# Phenotypical peculiarities and species‐specific differences of canine and murine satellite glial cells of spinal ganglia

**DOI:** 10.1111/jcmm.16701

**Published:** 2021-06-06

**Authors:** Bei Huang, Isabel Zdora, Nicole de Buhr, Annika Lehmbecker, Wolfgang Baumgärtner, Eva Leitzen

**Affiliations:** ^1^ Department of Pathology University of Veterinary Medicine Hannover Germany; ^2^ Center of Systems Neuroscience Hannover Germany; ^3^ Department of Biochemistry University of Veterinary Medicine Hannover Germany; ^4^ Research Center for Emerging Infections and Zoonoses (RIZ) University of Veterinary Medicine Hannover Germany

**Keywords:** canine, dorsal root ganglia, glutamine synthetase, Kir 4.1, murine, satellite glial cells, spinal ganglia

## Abstract

Satellite glial cells (SGCs) are located in the spinal ganglia (SG) of the peripheral nervous system and tightly envelop each neuron. They preserve tissue homeostasis, protect neurons and react in response to injury. This study comparatively characterizes the phenotype of murine (mSGCs) and canine SGCs (cSGCs). Immunohistochemistry and immunofluorescence as well as 2D and 3D imaging techniques were performed to describe a SGC‐specific marker panel, identify potential functional subsets and other phenotypical, species‐specific peculiarities. Glutamine synthetase (GS) and the potassium channel Kir 4.1 are SGC‐specific markers in murine and canine SG. Furthermore, a subset of mSGCs showed CD45 immunoreactivity and the majority of mSGCs were immunopositive for neural/glial antigen 2 (NG2), indicating an immune and a progenitor cell character. The majority of cSGCs were immunopositive for glial fibrillary acidic protein (GFAP), 2',3'‐cyclic‐nucleotide 3'‐phosphodiesterase (CNPase) and Sox2. Therefore, cSGCs resemble central nervous system glial cells and progenitor cells. SGCs lacked expression of macrophage markers CD107b, Iba1 and CD204. Double labelling with GS/Kir 4.1 highlights the unique anatomy of SGC‐neuron units and emphasizes the indispensability of further staining and imaging techniques for closer insights into the specific distribution of markers and potential colocalizations.

## 1 INTRODUCTION

Satellite glial cells (SGCs) of the sensory ganglia are a glial cell population of the peripheral nervous system (PNS) with diverse and remarkable features. Within the past two decades, SGCs have received increasing attention in a wide field of research. In addition to studying their morphological and functional qualities, their role in pathologic states and the development of neuropathic pain has been investigated.^1^ The sensory ganglia, including the trigeminal ganglion as well as the spinal ganglia (SG), also known as dorsal root ganglia (DRG), are part of the PNS and transmit sensory signals from the periphery towards the central nervous system (CNS).^2^ Sensory ganglia are mainly composed of neuronal somata, Schwann cells and SGCs. A unique anatomical feature of SGCs represents the tight glial sheath they form around neurons, a characteristic not seen in any other glial cell type of the CNS or PNS.^3^, ^4^ Typically, several SGCs enclose one neuronal soma as well as the initial portion of the axon.^3^, ^4^ The close morphological contact between SGCs and neurons already alludes to an intimate functional interdependence.^4^, ^5^, ^6^ The enveloping SGCs thus seem to possess a comparable role to astrocytes in the CNS in preventing direct contact between blood vessels and neurons.^4^, ^6^, ^7^, ^8^ A thin layer of connective tissue separates each neuron‐SGC unit. Sensory neurons do not form synapses to each other. However, intercellular communication is thought to be achieved by exchanging signals through and with SGCs. SGCs and neurons are believed to communicate via transmission of chemical substances such as ATP and Ca^2+^ as well as receptor‐dependent activation of intracellular pathways.^7^, ^9^, ^10^, ^11^ Beyond that, SGCs have proven to interconnect with each other through gap junctions.^12^, ^13^ SGCs display important modulatory and protective functions for controlling and maintaining the microenvironment of neurons, comparable to central glial cells. Generally, the response of SGCs to injury, for example to the peripheral nerve is multifaceted. For instance, murine and rat SGCs begin to proliferate, become hypertrophic and upregulate the expression of glial fibrillary acidic protein (GFAP) in pathologic conditions.^14^, ^15^, ^16^, ^17^ A recent study investigating transcriptional changes in SGCs following peripheral nerve injury demonstrates that SGCs are also engaged in ‘injury‐induced immune‐related processes in the DRG’.^18^ Furthermore, human trigeminal SGCs express a variety of Toll‐like receptors (TLR) and produce cytokines after stimulation with eligible TLR ligands. Thus, SGCs might also play an important role in triggering and managing the inflammatory response to pathogens.^19^

Interestingly, in vitro studies of SGCs indicate that they represent multipotent glial cells or may even display developmentally arrested Schwann cells.^20^, ^21^, ^22^ Moreover, SGCs seem to be susceptive to being differentiated towards specific phenotypes resembling that of oligodendrocytes, oligodendrocyte precursor cells and astrocytes, in vitro.^20^, ^22^, ^23^ These features could make SGCs promising candidates for further research in regeneration and reparation after CNS injury.

Overall, SGCs appear to be a plastic cell population with multiple functional roles. Although the interest in this cell population is growing, few studies have specifically dealt with canine SGCs (cSGCs), and current knowledge of cSGCs is scarce in comparison to murine (mSGCs) and rat SGCs.^23^, ^24^, ^25^ The dog is of particular interest as it represents a suitable translational large animal model for certain canine and human CNS diseases, including spinal cord injury. Dogs show comparable pathogenic mechanisms, lesion distribution and morphology as well as clinical manifestations.^26^, ^27^, ^28^ A better understanding of cSGCs and their potential regenerative properties will be beneficial for future applications in regenerative medicine. The goal of this study is to provide a detailed phenotypical analysis of cSGCs in direct comparison to mSGCs. Furthermore, the study aimed for an in‐depth characterization of the expression and anatomical localization of selected markers using different staining and visualization methods. Finally, it was aspired to investigate potentially different functional subsets of SGCs and at the same time for detecting possible interspecies differences.

## 2 MATERIALS & METHODS

### 2.1 Animals and tissue sampling

Cervical, thoracic and lumbar SG of six female and two male, adult C57BL/6 wildtype mice were harvested for this study. Likewise, cervical, thoracic and lumbar SG from two female and two male, adult Beagle dogs were used. Dogs were housed under standardized conditions, routinely checked and considered healthy when euthanized for purposes not related to this study. All SG were extracted post‐mortally and removed shortly after euthanasia. Mice were euthanized in compliance with the law of animal welfare approved by the commercial and veterinary affairs office of the state capital of Lower‐Saxony, Hannover (permission number: 42500/1H). SG were immediately stored on ice in phosphate‐buffered saline (PBS; pH = 7,1) containing 1% penicillin‐streptomycin (PS; Sigma‐Aldrich, Merck KGaA, Darmstadt, Germany) (canine SG) or directly transferred to 10% neutrally buffered formalin (murine SG) until further processing. For immunohistochemistry (IHC) and immunofluorescence (IF), SG were fixed in formalin for at least 12 hours, embedded in paraffin wax and cut into approximately 3 µm thick sections and subsequently mounted on SuperFrost‐Plus® slides (Thermo Fisher Scientific Inc, Fisher Scientific GmbH, Schwerte, Germany). Alternatively, fresh‐frozen tissue was collected using optimal cutting temperature (OCT) compound (Tissue‐Tek^®^ OCT^™^ Compound, Sakura, Alphen aan den Rijn, Netherlands) and snap‐freezing in liquid nitrogen. The fresh‐frozen, OCT‐embedded (FFOE) SG were cut into approximately 5 µm sections on a cryostat (Leica, CM1950), mounted on SuperFrost‐Plus® slides, fixed in acetone (Roth C. GmbH & Co. KG) for 10 minutes and stored at −80℃ until use for IF staining.

### 2.2 Immunohistochemistry

For the phenotypical characterization of cSGCs and mSGCs, formalin‐fixed, paraffin‐embedded (FFPE) sections were immunostained performing the avidin‐biotin‐peroxidase complex (ABC) method. The used primary antibodies including dilutions and antigen retrieval technique are listed in Table 1. Sections were deparaffinized in Roticlear® (Roth C. GmbH & Co. KG) and rehydrated with graded series of alcohols. Endogenous peroxidase was blocked using 0.5% H_2_O_2_ in 85% ethanol for 30 minutes at room temperature (RT). Antigen retrieval was achieved by boiling in citrate buffer (pH = 6.0) for 20 minutes in a microwave (800 W). Non‐specific binding sites were blocked with inactivated serum from the respective host species of the secondary antibody, followed by overnight incubation of the primary antibodies diluted in PBS and 1% bovine serum albumin (BSA) at 4℃. As negative controls, primary antibodies were replaced by inactivated serum accordingly. Sections were incubated with biotinylated secondary antibodies (1:200 in PBS; goat anti‐rabbit IgG; goat anti‐mouse IgG; Vector Laboratories) for 45 minutes at RT and subsequently treated with the avidin‐biotin‐peroxidase complex (Vectastain ABC Kit, PK 6100, Vector Laboratories) for 30 minutes at RT. The antigen‐antibody reaction was visualized by applying 3,3‐diaminobenzidine tetrahydrochloride (DAB, 0.05%, Sigma‐Aldrich Chemie GmbH) with addition of 0.03% H_2_O_2_. Finally, slides were counterstained with Mayer's haematoxylin (Roth C. GmbH & Co KG), dehydrated and mounted with ROTI® Histokitt II (Roth C. GmbH & Co KG).

**TABLE 1:** Primary antibodies used for immunohistochemistry (IHC) and immunofluorescence (IF)

Primary antibody specificity	Clonality	Source	Dilution					
			IHC‐FFPE		IF‐FFPE		IF‐FFOE	
			Dog	Mouse	Dog	Mouse	Dog	Mouse
CD107b	mc rat	MCA2293, Clone M3/84, Bio‐Rad Laboratories Inc,	‐	1:600	‐	‐	‐	1:200
CD204	mc mouse	MAB1710, Clone SRA‐E5, Abnova Corporation	1:500	‐	1:250	‐	‐	‐
CD45	mc rat	CD45 Monoclonal Antibody (30‐F11), eBioscience™, Invitrogen, Thermo Fisher Scientific	‐	1:400	‐	‐	‐	1:100
CNPase	mc mouse	MAB326, clone 11‐5B, Sigma‐Aldrich, Merck KGaA	1:100	1:100	1:100	1:100	‐	‐
GFAP	mc mouse	G3893, Sigma‐Aldrich, Merck KGaA, Darmstadt, Germany	1:800	1:800	1:400	1:400	‐	‐
GS	pc rabbit	PA5‐28940, Invitrogen, Thermo Fisher Scientific	1:4000	1:2000	1:2000	1:2000	‐	‐
Iba1	pc goat	011‐27991, FUJIFILM Wako Pure Chemical Corporation	‐	‐	1:400	1:400	‐	‐
Kir 4.1	pc rabbit	APC‐035, Alomone laboratories Ltd,	1:2000	1:4000	1:1000	1:2000	‐	‐
Kir 4.1‐AG	pc rabbit	APC‐035‐AG, Alomone laboratories Ltd,	‐	‐	‐	‐	1:200	1:500
Nestin	pc rabbit	AP 07829PU‐N, OriGene Technologies	1:500	1:500	‐	‐	‐	‐
NeuN	mc mouse	MAB377, Clone A60, Sigma‐Aldrich, Merck KGaA	‐	‐	1:800	1:800	‐	‐
NG2	pc rabbit	AB5320, Sigma‐Aldrich, Merck KGaA	‐	‐	‐	‐	‐	1:100
Periaxin	pc rabbit	HPA001868‐100UL, Sigma‐Aldrich, Merck KGaA	1:5000	1:5000	‐	‐	1:1000	1:1000
Sox2	mc rabbit	3579S, Cell Signaling Technology Inc	1:50	1:50	1:50	‐	‐	‐

Abbreviations: CNPase, 2′,3′‐cyclic‐nucleotide 3′‐phosphodiesterase; FFOE, fresh‐frozen, OCT‐embedded; FFPE, formalin‐fixed, paraffin‐embedded; GFAP, glial fibrillary acidic protein; GS, glutamine synthetase; Iba1, ionized calcium‐binding adapter molecule 1; Kir 4.1, inwardly rectifying potassium channel 4.1; mc, monoclonal; NG2, neural/glial antigen 2; pc, polyclonal; Sox2, sex determining region Y‐box 2.

For nestin (AP 07829PU‐N, OriGene Technologies), the two step IHC staining technique Dako EnVision®+ (Dako Envision+System‐HRP rabbit; Agilent Technologies Inc,) was used. After overnight incubation of the primary antibody, the Dako EnVision+System‐ HRP‐labelled polymer anti‐rabbit was added to the slides for 30 minutes at RT. Afterwards, slides were incubated with DAB with addition of 0.03% H_2_O_2_ for 5 minutes.

### 2.3 Immunofluorescence

Departing from the protocol for FFPE material indicated above, primary antibodies were diluted in PBS with addition of 1% BSA and 0.1% Triton‐X (PBST‐BSA; Triton® X‐100, Merck Millipore, Merck KGaA). After overnight incubation at 4℃, appropriate secondary antibodies (diluted 1:200 in PBST‐BSA; Cy^TM^‐2‐conjugated AffiniPure goat anti‐rabbit IgG (H + L); Cy^TM^‐3‐conjugated AffiniPure goat anti‐mouse IgG (H + L); Cy™3 AffiniPure Donkey Anti‐Goat IgG (H + L); Alexa Fluor® 488 AffiniPure Donkey Anti‐Rabbit IgG (H + L); Jackson ImmunoResearch Europe Ltd,) were applied for 1 hour at RT. Lastly, sections were counterstained with 0.01% Bisbenzimidin (diluted in Aqua bidestillata; bisBenzimide H 33 258, Merck KGaA, Darmstadt, Germany) for 10 minutes at RT and subsequently mounted with fluorescence mounting medium (Dako North America Inc.).

For FFOE tissue, acetone‐fixed sections were thawed at RT, washed with PBS and incubated with PBST for 30 minutes. Unspecific binding sites were blocked for 1 hour at RT as described above and again washed with PBST. Primary antibodies diluted in PBST‐BSA were applied and left on overnight at 4℃. For the double labelling of CD107b (MCA2293, Clone M3/84, Bio‐Rad Laboratories Inc,) and CD45 (30‐F11, eBioscience™, Invitrogen, Thermo Fisher Scientific) with Kir 4.1. (APC‐035, Alomone laboratories Ltd,), both primary antibodies were added simultaneously. Afterwards, sections were washed with PBST or PBS plus 1% Tween® 20 (SERVA Electrophoresis GmbH, Heidelberg, Germany; NG2 and CD45), respectively. Appropriate secondary antibodies (1:200 in PBST‐BSA; Cy^TM^‐2‐conjugated AffiniPure goat anti‐rabbit IgG (H + L); Cy^TM^‐3‐conjugated AffiniPure goat anti‐mouse IgG (H + L); Cy^TM^‐3‐conjugated AffiniPure goat anti‐rat IgG (H + L) Jackson ImmunoResearch Europe Ltd, Ely, UK) were applied for 1 hour at RT. Finally, sections were washed with PBST and double‐distilled water before being counterstained with 0,01% Bisbenzimidin (in double‐distilled water) and mounted with fluorescence mounting medium.

For double labelling of the directly labelled Kir 4.1 (APC‐035‐AG, Alomone laboratories Ltd,) with NG2 (AB5320, Sigma‐Aldrich, Merck KGaA) or periaxin (HPA001868‐100UL, Sigma‐Aldrich, Merck KGaA), non‐labelled antibodies were initially incubated for 24 hours. After incubation with the appropriate secondary antibodies on the next day, the directly labelled Kir 4.1 antibody was incubated for another 24 hours.

### 2.4 Laser scanning confocal microscopy and 3D reconstruction

In order to further illustrate and confirm the localization of selected markers, confocal recordings of particular IF double labelling were generated. Laser scanning images were captured with a Leica TCS SP5 AOBS confocal inverted‐base fluorescence microscope (Leica Microsystems, Bensheim, Germany) with a HCX PL APO lambda blue 40x 1.25 oil immersion objective. For each double labelling, the laser settings were adjusted according to the appropriate controls. For the 3D images and movies, a series of optical sections (z‐stacks) were collected and analysed with LAS X 3D version 3.1.0 software from Leica. Z‐stack pictures were used and the background set to black by standard software settings.

### 2.5 Picture analysis

Images of immunohistochemical and‐fluorescence staining were captured with a BZ‐9000E microscope (Keyence Deutschland GmbH). Selected sections were also analysed in 3D‐reconstructed images of confocal laser microscopy.

### 2.6 Canine SG

For antibodies creating a distinct signal in IHC of FFPE material, three SG of each of the four dogs were analysed. For each antibody, a maximum of ten randomly selected high power fields (40X) were evaluated. Immunopositive and immunonegative SGCs within the pictures as well as the number of associated neurons were counted manually using Fiji Is Just ImageJ software.^29^

For CD204 and Iba1, IF double labelling with GS of one representative SG of each dog was performed in order to rule out false‐positive results. Prior to quantitative analysis, exemplary sections were investigated using laser scanning confocal microscopy. A maximum of ten pictures per SG was examined, and the percentage of immunopositive SGCs was calculated.

### 2.7 Murine SG

IHC staining of FFPE SG of four mice was analysed by manually counting all visible neurons and associated immunonegative and immunopositive SGCs in 40X magnification. For exclusion of false‐positive results and examination of formalin sensitive epitopes, a subset of antibodies (CD107b, CD45, periaxin, NG2) was used in IF using one representative SG per mouse. For CD45 and NG2, double labelling with the SGC‐specific marker Kir 4.1 was performed. Furthermore, prior to quantification, 3D‐reconstructed images of these staining were analysed to substantiate results from 2D images.

### 2.8 Colocalization analysis

To further substantiate a potential overlap of selected markers, that could represent potential functional subsets (NG2 with Kir 4.1 and CD45 with Kir 4.1), the EzColocalization plugin for ImageJ (version 1.53c; http://imagej.nih.gov.ij/) was exemplary applied to selected z‐stack images captured with a confocal microscope.^30^ Furthermore, z‐stack images of a marker anticipated not to co‐localize with SGC markers (CD204 and GS) were included as an internal control. All single images from z‐stacks were investigated using Spearman's rank correlation coefficient (SRCC) for multicolour image correlation via ranking of pixel intensity values (−1 = perfect negative association of ranks; 0 = no association; 1 = perfect positive association of ranks). Moreover, Manders’ Colocalization Coefficient (MCC), a suitable measure to quantify colocalization in biological microscopy, was applied (0 = complete anti‐colocalization; 1 = complete colocalization).^31^, ^32^ Unlike MCC, the SRCC does not evaluate the degree of overlap between both channels. SRCC acts as a predictor of signal intensities within the same pixel, which can be, inter alia, used as an indicator of potential functional correlation.^33^

## 3 RESULTS

A representative marker panel was chosen from recent literature to comparatively analyse the expression pattern of SGCs within the SG of mice and dogs. For further analysis of potential functional subsets, these markers were subdivided into four groups, (a) SGC‐specific markers (GS, Kir 4.1), which have been described to be expressed in mSGCs^6^, ^34^, ^35^, ^36^ (b) glial cell markers (periaxin, GFAP, CNPase), that could on the one hand indicate a central glial cell character and on the other hand a peripheral glial cell character with potential regenerative benefits, (c) immune cell markers (CD45, CD107b, CD204, Iba 1) due to a proposed engagement of SGCs in immune cell processes ^18^ and (d) neural progenitor markers (NG2, nestin, Sox2) in order to investigate a potential multipotent character of SGCs.^20^, ^22^ The results of the quantitative analysis of all applied markers (Table 1) are depicted in Table 2.

**TABLE 2:** Quantitative analysis of murine and canine spinal ganglia (SG)

Thematic grouping	Antibody specificity	Species	Material and staining technique used for quantitative analysis	Number of animals analysed	Total number of SG analysed/stained	Mean percentage of immunopositive SGCs
SGC‐specific markers	glutamine synthetase (GS)	Dog	FFPE, IHC (ABC method)	4	12	97.8%
		Mouse		4	36	83.4%
	Kir 4.1	Dog	FFPE, IHC (ABC method)	4	11	95.2%
		Mouse		4	45	94.5%
Glial cell markers	**glial fibrillary acidic protein (GFAP)**	Dog	FFPE, IHC (ABC method)	4	12	98.9%
		Mouse		4	36	0%
	periaxin	Dog	FFPE, IHC (ABC method)	4	12	0.6%
		Mouse	FFOE, IF	4	4	0%
	**2',3'‐cyclic‐nucleotide 3'‐phosphodiesterase (CNPase)**	Dog	FFPE, IHC (ABC method)	4	12	97.0%
		Mouse	FFPE, IHC (ABC method)	4	33	0%
Immune cell markers	Iba 1	Dog	FFPE, IF	4	4	0.7%
		Mouse	FFPE, IHC (ABC method)	4	37	0%
	CD107b	Mouse	FFOE, IF	4	4	0%
	CD204	Dog	FFPE, IF	4	4	0%
	CD45	Dog	excluded	excluded	excluded	excluded
		Mouse	FFOE, IF	4	4	73.9%
Neural progenitor markers	**Sox2**	Dog	FFPE, IHC (ABC method)	4	12	96.6%
		Mouse		4	46	0%
	nestin	Dog	FFPE, IHC (EnVision+ method)	4	12	1.6%
		Mouse		4	46	0%
	neural/glial antigen 2 (NG2)	Dog	excluded	excluded	excluded	excluded
		Mouse	FFOE, IF	4	4	55.6%

Note

The mean percentage of immunopositive satellite glial cells (SGCs) for glial fibrillary acidic protein (GFAP), 2′,3′‐cyclic‐nucleotide 3′‐phosphodiesterase (CNPase) and Sox2 showed an exceptional difference between the two species (highlighted in grey and in bold).

Abbreviations: IF, immunofluorescence; IHC, immunohistochemistry.

### 3.1 SGC‐specific markers: GS and Kir 4.1

**FIGURE 1:**
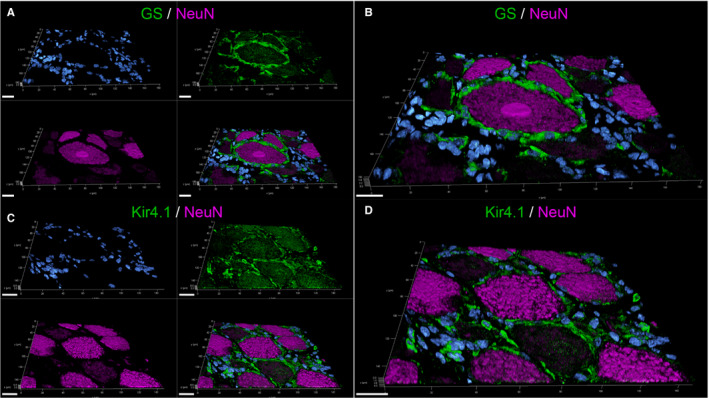
3D‐reconstructed confocal laser images of formalin‐fixed paraffin‐embedded canine spinal ganglia (A‐D): Double labelling with glutamine synthetase (GS; green; A, B) or inwardly rectifying potassium channel Kir 4.1 (green; C, D), respectively, and the neuronal marker NeuN (magenta). Nuclei are counterstained with bisbenzimide (blue). The zoomed in pictures (B, D) show that GS‐, respectively, Kir4.1‐positive satellite glial cells (SGCs) tightly envelop NeuN‐positive neurons. For GS/NeuN staining (A‐B), 32 z‐stack frames (5.2 µm total size; approx. 0.16 µm steps) and for Kir4.1/NeuN staining (C‐D), 31 z‐stack frames (5.0 µm total size; approx. 0.16 µm steps) were collected. Scale bars: 20 µm. A movie of 3D confocal reconstructions is provided in Video [Link], [Link] and Video [Link], [Link]

**FIGURE 2:**
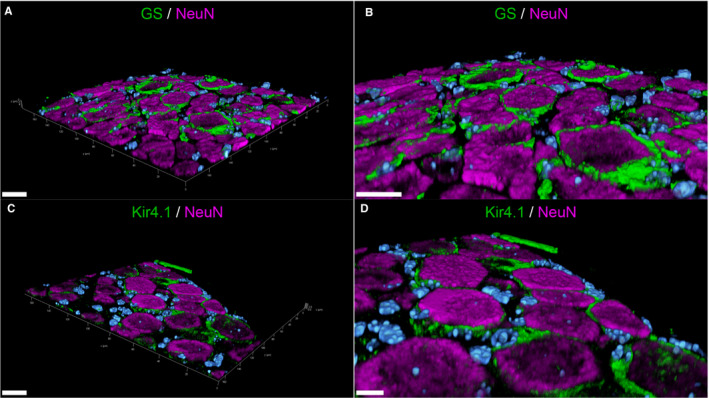
3D‐reconstructed confocal laser images of formalin‐fixed paraffin‐embedded murine spinal ganglia (A‐D): Double labelling with glutamine synthetase (GS; green; A, B) or inwardly rectifying potassium channel Kir 4.1 (green; C, D), respectively, and the neuronal marker NeuN (magenta). Nuclei are counterstained with Bisbenzimide (blue). GS‐ / Kir4.1‐positive satellite glial cells (SGCs) form a tight sheath around the neuronal bodies. The zoomed in images (B, D) clearly illustrate the close contact between SGCs and neurons. For GS/NeuN staining (A‐B), 39 z‐stack frames (6.4 µm total size; approx. 0.16 µm steps) and for Kir4.1/NeuN staining (C‐D), 39 z‐stack frames (4.7 µm total size; approx. 0.12 µm steps) were obtained. Scale bars: 20 µm (A, B, C); 10 µm (D). A movie of 3D confocal reconstructions is provided in Video [Link], [Link] and Video [Link], [Link]

IHC and IF staining revealed GS to be a SGC‐specific marker not only in murine but also canine SG. In IHC, 83.42% of mSGCs and 97.84% of cSGCs were immunopositive for GS. The 3D‐reconstructed images of immunofluorescence double labelling of GS and the neuronal marker NeuN (neuronal nuclei, hexaribonucleotide binding protein‐3; marker of mature neurons) corroborate these results (Figure 1A,B and Figure 2A,B; a movie of 3D confocal reconstructions of Figure 1A,B is provided in Video S1; a movie of 3D confocal reconstructions of Figure 2A,B is provided in Video S2). A rim of GS‐positive SGCs surrounds the neuronal somata, while other cells of the SG show no immunoreaction for GS. Consequently, GS was used in the ensuing double labelling with other selected markers.

Similar to GS, the vast majority of the investigated mSGCs (94.52%) and cSGCs (95.24%) were immunopositive for the inwardly rectifying potassium channel Kir 4.1. 3D‐reconstructed images of murine and canine SG with Kir 4.1 and NeuN fortify these results, too (Figure 1C,D and Figure 2C,D; a movie of 3D confocal reconstructions of Figure 1C,D is provided in Video S3; a movie of 3D confocal reconstructions of Figure 2C,D is provided in Video S4). Kir 4.1‐positive SGCs closely enwrap NeuN‐positive sensory neurons. Cells in between neuron‐SGC units show no immunoreaction for Kir 4.1. Therefore, Kir 4.1 was considered a SGC‐specific marker in murine and canine SG and used for double labelling with other markers along with GS.

In addition, an antibody specifically targeting an extracellularly located epitope of Kir 4.1 (APC‐165, Alomone laboratories Ltd.) was also found in mSGCs and cSGCs (data not shown). This is particularly interesting for prospective in vitro studies, because extracellularly located epitopes can be targeted in order to separate the desired cell population from others.

### 3.2 Glial cell markers: GFAP, periaxin, CNPase

**FIGURE 3:**
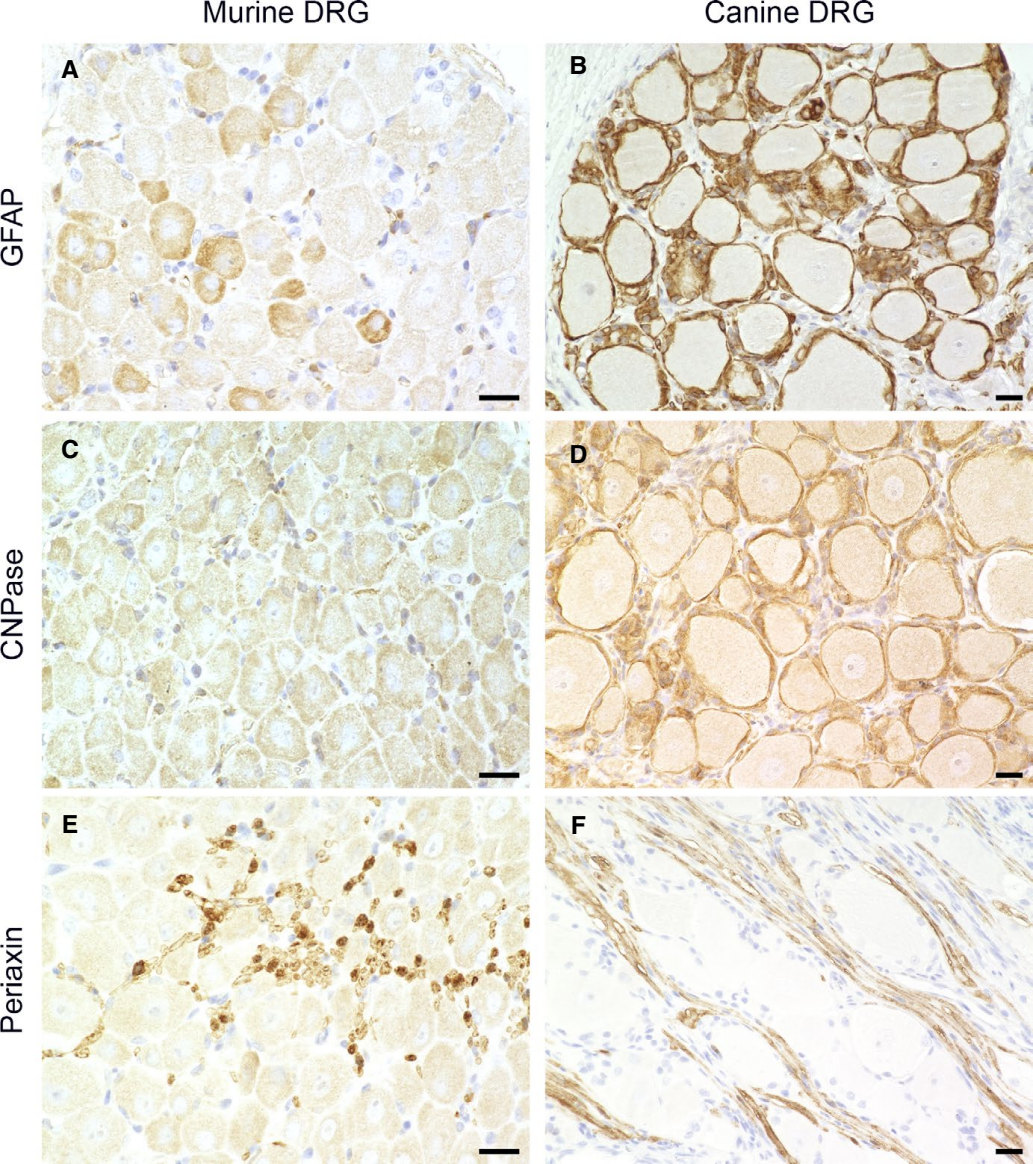
Immunohistochemistry of formalin‐fixed, paraffin‐embedded murine (A, C, E) and canine spinal ganglia (B, D, F): Staining for glial fibrillary acidic protein (GFAP; A, B), 2′,3′‐cyclic‐nucleotide 3′‐phosphodiesterase (CNPase; C, D) and periaxin (E, F). None of the murine satellite glial cells (SGCs) show immunoreactivity for GFAP (A) and CNPase (C), while the majority of canine SGCs are immunopositive for GFAP (B) and CNPase (D). In both species, SGCs showed no immunoreactivity for periaxin in contrast to Schwann cells (E, F). Scale bars: 20 µm

In the performed immunohistochemical staining, 0% of mSGCs, but 98.87% of cSGCs, stained positive for the intermediate filament III protein GFAP (Figure 3; Figure S1), a common marker of mature astrocytes. Immunofluorescence double labelling of cSGCs with GFAP and GS confirmed the colocalization of both markers.

Neither mSGCs nor cSGCs showed an immunoreaction for periaxin. However, myelinating Schwann cells of adjacent axons consistently stained positive for periaxin (Figure 3). The afferent and efferent nerve fibres are in direct vicinity to the neuronal somata and their respective SGC sheaths, which posed the risk of mistaking Schwann cells for SGCs and vice versa. Immunofluorescence double labelling of periaxin with the SGC‐specific marker Kir4.1 was performed in order to rule out false‐positive results (Figure S2). In this study, no mSGCs, but a majority (97.01%) of cSGCs, were immunopositive for CNPase (Figure 3).

### 3.3 Immune cell markers: Iba1, CD107b, CD204, CD45

**FIGURE 4:**
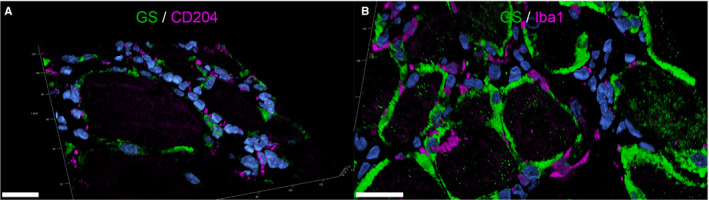
3D‐reconstructed confocal laser images of double labelling of formalin‐fixed paraffin‐embedded canine spinal ganglia: Double labelling with the macrophage markers CD204 (magenta; A) and Iba1 (magenta; B) and the satellite glial cell (SGC)‐specific marker glutamine synthetase (GS; green). Nuclei are counterstained with bisbenzimide (blue). Cells immunopositive for CD204 and Iba1 are in very close vicinity to GS‐positive SGCs. However, there is no colocalization of GS and CD204/Iba1 detectable in SGCs. Therefore, detected cells presumably represent tissue resident macrophages. For GS/CD204 co‐labelling (A), 39 z‐stack frames (6.38 µm total size; approx. 0.16 µm steps) and for GS/Iba1 co‐labelling (B), 34 z‐stack frames (5.54 µm total size; approx. 0.16 µm steps) were acquired. Scale bars: 20 µm. A movie of 3D confocal reconstructions is provided in Video [Link], [Link] and Video [Link], [Link]

None of the mSGCs showed immunoreactivity for Iba1. For canine tissue, IF double labelling of Iba1 with GS were performed. 2D images did not allow determining whether SGCs or immune cells in close vicinity were immunopositive for Iba1. Interestingly, the subsequent 3D reconstruction of confocal z‐stacks indicates that the majority of cSGCs lacked Iba1 expression. In fact, it seems that resident macrophages next to SGCs are the main source of the obtained signals (Figure 4; a movie of 3D confocal reconstructions of Figure 4 B is provided in Video S5).

In the canine SG, a limited number of cells expressed CD204. Again, IF double labelling with GS as well as confocal microscopy of a representative SG revealed that most likely resident macrophages in close vicinity to SGCs were immunopositive for CD204. No SGCs showed co‐labelling of GS and CD204 (Figure 4; a movie of 3D confocal reconstructions of Figure 4 A is provided in Video S6).

0% of the investigated mSGCs showed immunoreaction for CD107b (Figure S3).

**FIGURE 5:**
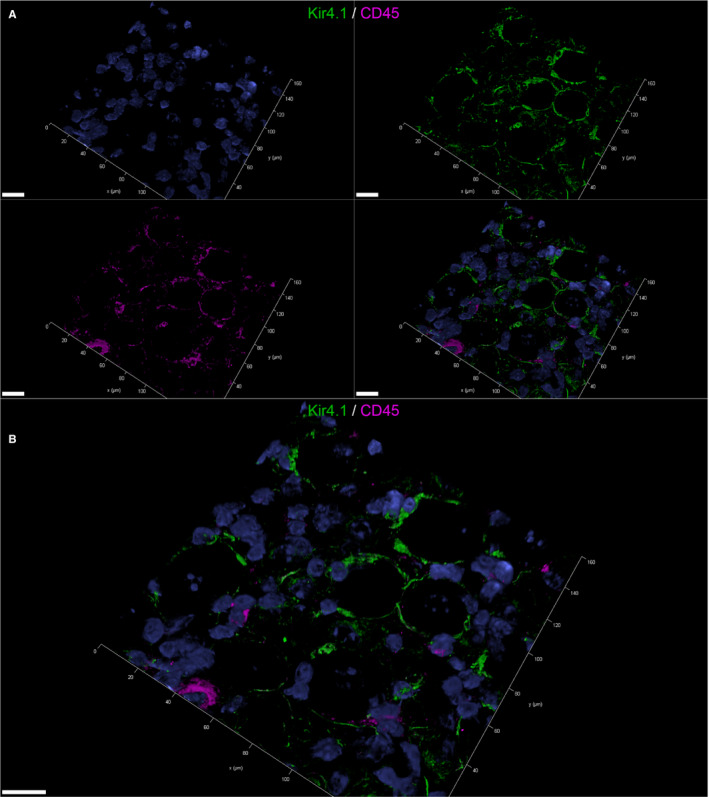
3D‐reconstructed confocal laser images showing double labelling of fresh‐frozen, OCT‐embedded murine spinal ganglia: Double labelling with the pan‐leukocyte marker CD45 (magenta) and the satellite glial cell (SGC)‐specific inwardly rectifying potassium channel Kir4.1 (green) (A, B). Nuclei are counterstained with bisbenzimide (blue). In the split channel image (A), CD45 labelling of Kir4.1‐positive SGCs can be appreciated. 73.89% of murine satellite glial cells (SGCs) were immunopositive for CD45. The merged image (B) also shows some CD45‐positive cells that do not double label with Kir4. These cells were considered tissue resident immune cells. 41 z‐stack frames (6.7 µm total size; approx. 0.16µm steps) were collected. Scale bars: 20 µm. A movie of 3D confocal reconstructions is provided in Video S7

73.89% of mSGCs were immunopositive for CD45 in IF staining of FFOE material (Figure 5; a movie of 3D confocal reconstructions of Figure 5 is provided in Video S7). Using splenic tissue as positive control, anti‐CD45 antibody staining pattern observed in canine tissue did not reflect the organ‐typical structure and was therefore considered to have low sensitivity in canine tissue.

### 3.4 Neural progenitor markers: NG2, Sox2, nestin

**FIGURE 6:**
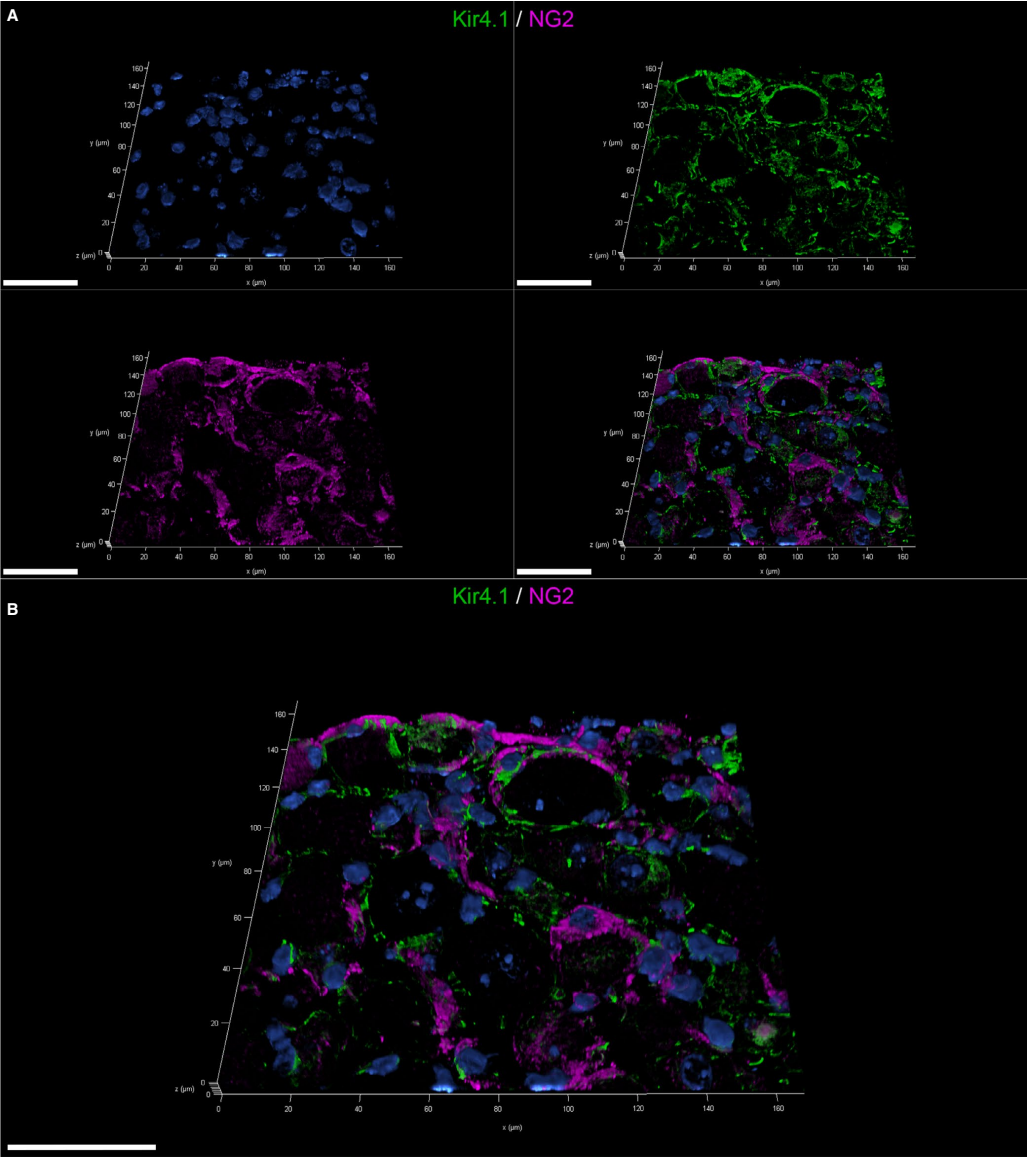
3D‐reconstructed confocal laser images showing double labelling of fresh‐frozen, OCT‐embedded murine spinal ganglia: Double labelling with the neural/glial antigen 2 (NG2; magenta) and the satellite glial cell (SGC)‐specific inwardly rectifying potassium channel Kir4.1 (green) (A, B). Nuclei are counterstained with bisbenzimide (blue). The split channel image (A) shows the colocalization of the NG2 signal in Kir4.1‐positive SGCs. More than half of murine SGCs were immunopositive for NG2. 40 z‐stack frames (6.5 µm total size; approx. 0.16 µm steps) were collected. Scale bars: 50 µm. A movie of 3D confocal reconstructions is provided in Video S8

**FIGURE 7:**
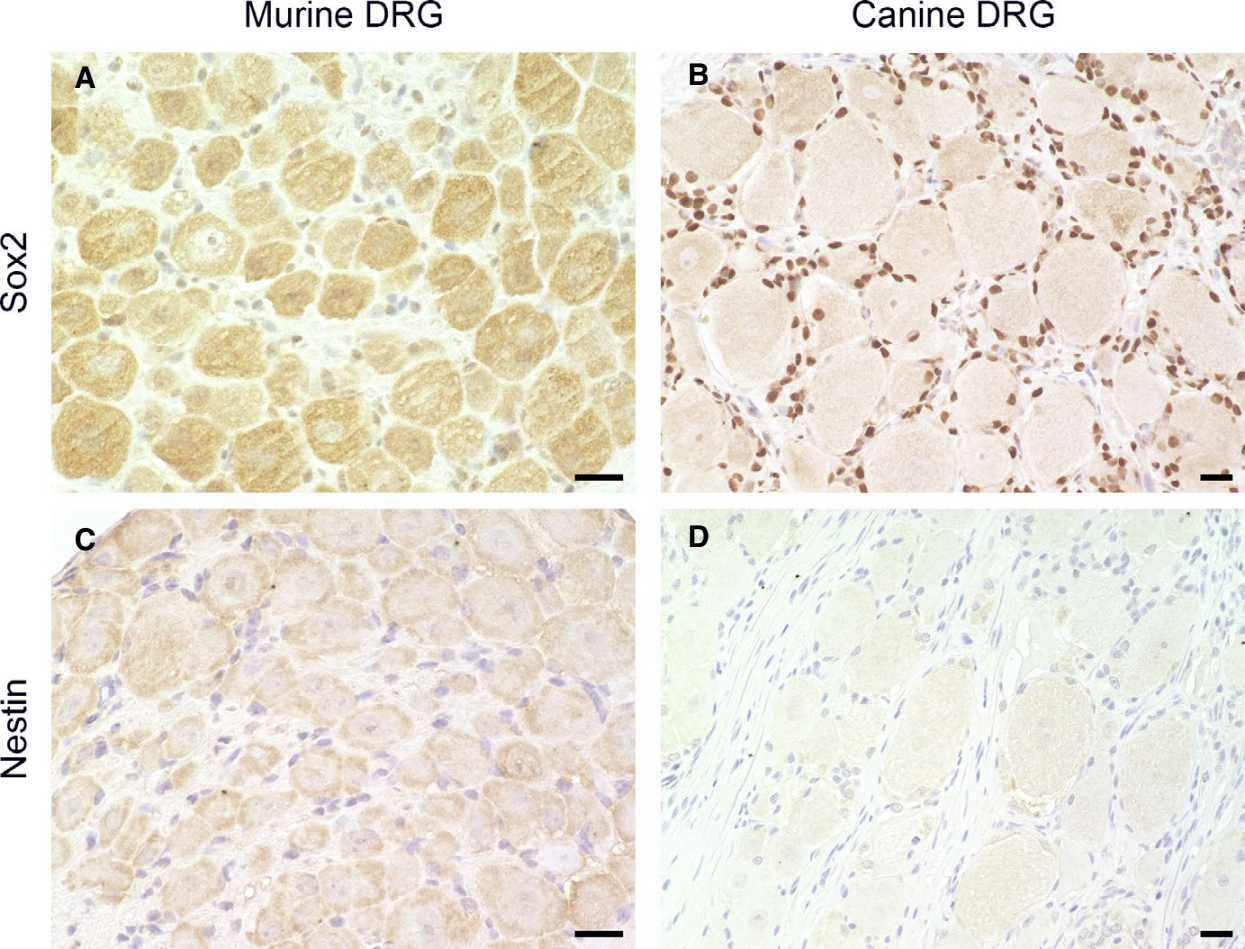
Immunohistochemistry of formalin‐fixed, paraffin‐embedded murine (A, C) and canine spinal ganglia (B, D): Staining for the transcription factor Sox2 (A, B) and nestin (C, D). Most of the canine satellite glial cells (SGCs) are immunopositive for Sox2 with a clear nuclear signal (B), while none of the murine SGCs show immunoreactivity (A). In both species, none of the SGCs showed an immunoreaction for nestin (C, D). Scale bars: 20 µm

55.6% of the analysed mSGCs expressed NG2. The 3D‐reconstructed image (Figure 6; a movie of 3D confocal reconstructions of Figure 6 is provided in Video S8) confirms the co‐labelling of mSGCs with the SGC‐specific marker Kir 4.1 and NG2. IF staining of FFOE material created a more distinct staining pattern compared to IHC of FFPE material. Hence, FFOE tissue processing proved to be best suitable for this antibody. The anti‐NG2 antibody of this study did not work appropriately on canine tissue and was therefore excluded for this species. In the present study, 0% of mSGCs and 96.63% of cSGCs stained positive for Sox2 (Figure 7). IHC and IF produced a clear nuclear signal in Sox2 positive cSGCs (Figure 7; Figure S1). In IHC, 0% of the investigated mSGCs and cSGCs showed an immunoreaction for nestin (Figure 7).

### 3.5 Colocalization analysis

Exemplary evaluation of confocal images of murine SG stained with Kir 4.1 and CD45 revealed a median SRCC of 0.394. This may indicate a positive—although not very pronounced—relationship of signal intensities, also graphically depicted within the associated metric matrix (Figure S4). Moreover, a median MCC of 0.778 for channel 1 (M1; fraction of Kir 4.1 containing CD45) and a median MCC of 0.489 for channel 2 (M2; fraction of CD45 containing Kir 4.1) could be detected. These results indicate a moderate to strong colocalization (more than 75% of objects positive for Kir 4.1 co‐localize with CD45) between Kir 4.1 and CD45 (Figure S4). Otherwise, approximately half of the objects positive for CD45 co‐localize with Kir 4.1 signals. Similarly, the analysis of Kir 4.1 and NG2 revealed a median SRCC of 0.572. This also indicates a moderately positive relationship between signal intensities. Markers show a median MCC of 0.857 for channel 1 (M1; fraction of Kir 4.1 containing NG2) and a median MCC of 0.759 for channel 2 (M2; fraction of NG2 containing Kir 4.1) (Figure S5), indicating a moderate to high level of colocalization in both directions.

Determination of the colocalization of GS and the macrophage marker CD204 in canine SG revealed a median SRCC of −0.003, indicating lack of association. At the same time, a median MCC of 0.271 for channel 1 (M1; fraction of GS containing CD204) and a median MCC of 0.008 for channel 2 (M2; fraction of CD204 containing GS) was observed. Especially, the M2 value suggests that a colocalization for these markers is highly unlikely (Figure S6).

## 4 DISCUSSION

Most of the recent studies focus on the phenotype of SGCs in an activated state, for example in the context of pain and nociception in response to injury. The aim of this study was to comparatively characterize mSGCs and cSGCs. Furthermore, the goal was to identify different subsets among SGCs that could be indicative of distinct functional aspects of SGCs.

### 4.1 GS and Kir 4.1 represent SGC‐specific markers in mice and dogs

Several studies have characterized GS as an SGC‐specific marker in murine and rat sensory ganglia that identifies SGCs in situ and *in vitro*.^6^, ^9^, ^35^, ^36^, ^37^, ^38^, ^39^, ^40^, ^41^ GS catalyses the conversion of the excitatory neurotransmitter glutamate to glutamine^42^ and is expressed by SGCs together with glutamate receptors and glutamate transporters.^43^ In the CNS, astrocytes represent the main glial cell population expressing GS.^44^ Interestingly, this study demonstrates that the majority of mSGCs and also the majority of cSGCs show GS‐immunoreactivity. This again indicates that GS represents a SGC‐specific marker regardless of the species investigated. Moreover, it underlines the prominent role of SGCs in regulating microenvironmental conditions and therefore contributing to neuron protection, comparable to CNS glial cells. While earlier studies partly hypothesized a lower percentage of GS‐positive cSGCs,^23^ double labelling and confocal microscopy revealed nearly 100% positivity for this marker. The use of different, potentially less sensitive antibodies, differences in tissue removal, processing and staining technique are among possible explanations for variable staining results. Moreover, variations in GS expression of SGCs have been mentioned before.^45^ Based on the results obtained from this study, GS can be used as a SGC‐specific marker not only in murine but also canine SG.

The inwardly rectifying potassium channel Kir 4.1 is responsible for K^+^ buffering, which regulates excitability of neurons, too.^46^ In the CNS, this subunit of potassium channels is again mainly expressed by astrocytes.^46^ Previous studies investigated the expression of Kir 4.1 by SGCs and its functional significance.^34^, ^47^, ^48^ In response to injury to the SG or the peripheral nerve, Kir 4.1 expression was significantly reduced leading to an increased excitability of neurons.^47^, ^49^, ^50^ It is assumed that Kir 4.1 is the main channel responsible for potassium influx and hence regulation of extracellular potassium concentrations in the SG.^34^ In this study, the majority of mSGCs expressed Kir 4.1. Interestingly, cSGCs consistently expressed Kir 4.1, too, which has not been described yet. This indicates a similar role of cSGCs in regulation of potassium concentration and therefore excitability of sensory neurons. Altogether, Kir 4.1 is considered a highly suitable SGC‐specific marker in the SG of both species. Moreover, GS and Kir 4.1 are also described to be specifically expressed by human SGCs of SG.^51^

### 4.2 Glial cell characteristics of SGCs

GFAP is involved in the structure and function of the cytoskeleton and therefore also in cell motility and migration. In astrocytes, an increased expression of GFAP indicates an activated state and plays an important role in the formation of the thickened and elongated processes.^52^ Similarly, GFAP expression is upregulated in activated, injured murine and rat SGCs of sensory ganglia, while it is often below detectable level in a non‐activated state.^16^, ^35^, ^53^, ^54^, ^55^ In contrast to mSGCs, the majority of cSGCs within this study expressed GFAP, which is in accordance with previously published data.^23^ It can be hypothesized that adult cSGCs possess a phenotype, which more closely resembles that of CNS glial cells, especially astrocytes. However, in spite of the high percentage of GFAP‐positive cSGCs, it should be kept in mind that—especially with regard to identification and purification of cells during in vitro studies—not only cSGCs, but also non‐myelinating Schwann cells express GFAP.^56^, ^57^ Therefore, GFAP did not qualify as an SGC‐specific marker in SG.

Some studies propose that cultured SGCs might be capable of axonal myelination.^20^, ^21^ However, little is known about the potential expression of myelin‐associated proteins in adult SGCs of dogs and mice. Since the myelin protein periaxin, a myelinating Schwann cell marker,^58^ was not detectable in mSGCs and cSGCs, further studies are needed to investigate, whether SGCs might upregulate and express periaxin, for example in response to injury to the PNS. CNPase is a membrane‐anchored enzyme primarily expressed by oligodendrocytes and represents the most abundant protein of the non‐compact myelin sheath in the CNS.^59^ Reports about CNPase expression in SGCs are limited. It is described that rat SGCs show almost no immunoreactivity for CNPase in a non‐activated state, but an increased expression of CNPase after spinal nerve ligation.^60^ The lack of CNPase expression in non‐activated rat SGCs resembles the results of mSGCs of the present study. Whether mSGCs will upregulate CNPase in consequence of injury needs to be further investigated. In contrast, the majority of cSGCs were immunopositive for CNPase, which meets the results of previous investigations.^23^ This might indicate that in dogs, CNPase is necessary for a sufficient interaction between SGCs and sensory neurons. Together with the strong GFAP expression, this could suggest a more prominent glial cell character of cSGCs and allude to different functions of mSGCs and cSGCs.

Furthermore, the ensuing aim is to investigate whether cSGCs and mSGCs can differentiate into cells in vitro with oligodendrocytic and/or Schwann cell characteristics with respect to, for example, the ability to myelinate axons. This could make them interesting candidates for transplantation studies with the objective of improved nerve regeneration and improved remyelination within CNS and PNS.

### 4.3 MSGCs and cSGCs do not exhibit macrophage‐related markers (Iba1, CD204, CD107b), but mSGCs display a subset of cells positive for common leukocyte antigen (CD45)

It has been proposed that SGCs influence the immune system or even display an immune cell character themselves. A recent study investigated the transcriptome of mSGCs.^18^ Genes linked to the immune system were enriched. Therefore, it was hypothesized that SGCs might interact with the immune system and activate or influence immune cell migration after nerve injury. Interestingly, current results indicate the presence of a subset of CD45^+^ mSGCs. CD45, also known as leukocyte common antigen, is a transmembrane protein with tyrosine phosphatase activity and serves as a pan‐leukocyte marker. It has been reported that SGCs of human trigeminal ganglia also express CD45 as well as several other macrophage markers and an immature myeloid dendritic cell marker.^61^

While no SGCs showed immunoreactivity for macrophage markers (Iba1, CD204 and CD107b), several immune cells, presumably resident macrophages, were scattered in between and found in very close contact to neuron‐SGC units. In immunohistochemistry, this harboured the risk of false‐positive results. The difficulty of identifying and distinguishing cells in the SG from each other was mentioned before.^18^, ^45^ To circumvent this pitfall, IF double labelling of questionable markers with Kir 4.1./GS was performed and analysed with confocal laser scanning microscopy. This again highlighted the fact that identifying and distinguishing cells in the SG from each other can be difficult when relying on one visualization method or on cell morphology only.

### 4.4 Expression of neural progenitor markers could indicate a potential regenerative capacity of SGCs

SGCs are derived from neural crest stem cells.^62^, ^63^ There is evidence that SGCs might retain stem cell characteristics in adult animals and are capable of dedifferentiation under certain conditions. The transcription factor Sox2 governs neural differentiation and sustains the self‐renewal of neural progenitor stem cells.^64^ High Sox2 reactivity was found in the SGCs of adult rat SG ^65^ and of young adult C57BL/6 mice.^36^ Furthermore, an increase in the expression of nestin was described under chronic pain conditions in mSGCs.^36^ In human adult trigeminal ganglia, nestin expression by SGCs has been described, too.^66^ Nestin is an intermediate filament, part of the cytoskeleton, and can be identified in a variety of cell types and stages including neural stem and progenitor cells.^67^ The results of the present studies did not detect immunoreactivity for Sox2 or nestin in adult mSGCs. Conversely, but in accordance with previous investigations, the majority of investigated adult cSGCs stained positive for Sox2 in SG.^23^ This could indicate that this cell population retains stem cell characteristics in adult dogs. It needs to be further elucidated, whether this could be a sign of a multipotent character of cSGCs and if they could function as a source of regenerative potential.

NG2 is also known as chondroitin sulphate proteoglycan 4 (CSPG4). Among other cells, oligodendrocyte precursor cells (OPCs), which are also called ‘NG2‐glia’, express this integral membrane protein. OPCs can differentiate into mature oligodendrocytes.^68^ However, many also remain in their immature state and represent a life‐long pool of adult progenitor cells.^69^ Interestingly, an NG2/GS‐positive subpopulation of adult mSGCs was identified, which could represent a functional subset with potential regenerative capacities among this cell population, too. In previous studies, SGCs from embryonic and post‐natal rat SG differentiated into cells resembling the phenotype of oligodendrocytic precursor cells positive for NG2 and platelet‐derived growth factor receptor‐α (PDGFRα).^20^ In another study, SGCs of lumbar rat SG also expressed NG2.^70^

However, NG2‐expression in adult mSGCs could represent a transient occurrence depending on the developmental state like it has been described in rats, too.^20^

In summary, this study clearly demonstrates the influence of tissue processing as well as different visualization techniques on SGC analysis. Choosing the most suitable technique not only minimizes the risk of false‐positive results but also reveals closer insights into the specific distribution of markers. Moreover, this study introduces GS and Kir 4.1 to be SGC‐specific markers not only in murine but also canine SG. These markers could be of special interest for targeted and effective in vitro cell isolation and purification. Interestingly, subsets of mSGCs immunopositive for CD45 or NG2 were found. This might indicate the existence of functional subgroups with immunological and/or progenitor cell properties within the population of mSGCs. The evaluation of the confocal z‐stack images of murine SG using Kir 4.1 combined with CD45 or NG2 further consolidates the impression of partial colocalization of these markers within SGCs. The results of the additionally analysed control section using a marker anticipated to be only located in close proximity to SGCs (CD204; macrophages) further substantiate the results. However, it needs to be mentioned that the values obtained are only able to describe a direct overlap of the channel signals. Therefore, the investigation might be restricted to the detection of real spatial colocalization. This comprises the risk of an underestimation of signals that are located within the same cell but within different compartments. Furthermore, obtained results presented within this study have been generated from a limited amount of data. Therefore, they should be interpreted as indicators towards the actual degree of colocalization. A more profound assertion would require additional experiments including an adjusted study design. In addition, evaluation whether SRCC values could hint towards an interrelationship between both markers or simply originate from physical proximity during intracellular transporting or artificial signal overlap would also require further investigations. However, SRCC and MCC values are well suited to obtain a first impression of quantity and quality of colocalization using selected markers, as shown for CD45 and NG2 in mSGCs.

There was a striking difference in the expression of GFAP, CNPase and Sox2 between mSGCs and cSGCs, which could hint towards a different function and developmental stage of SGCs with cSGCs exhibiting a more pronounced glial differentiation. Whether the observed species‐specific phenotypical peculiarities will change under pathological conditions and whether some of the features could be harnessed to use a potential regenerative capacity of SGCs needs to be further evaluated. A more in‐depth understanding of cSGCs is of particular value, since dogs represent important companion animals that also suffer from neurodegenerative diseases within CNS and PNS. Research on cSGC on the one hand addresses the growing need of regenerative therapeutic approaches in veterinary medicine. On the other hand, dogs also represent a suitable translational animal model for comparable human diseases, for example spinal cord injury.^26^, ^27^ In conclusion, SGCs represent a fascinating cell population that expresses a wide variety of interesting markers. These features make them attractive candidates for ensuing in vitro studies and research addressing regenerative processes post‐injury to, for example, the CNS/PNS in particular.

## CONFLICT OF INTEREST

The authors declare no conflict of interest.

## AUTHOR CONTRIBUTIONS

**Bei Huang:** Conceptualization (equal); Investigation (equal); Methodology (equal); Visualization (equal); Writing‐original draft (equal); Writing‐review & editing (equal). **Isabel Zdora:** Conceptualization (equal); Investigation (equal); Methodology (equal); Visualization (equal); Writing‐original draft (equal); Writing‐review & editing (equal). **Nicole de Buhr:** Investigation (supporting); Visualization (equal); Writing‐review & editing (equal). **Annika Lehmbecker:** Conceptualization (equal); Writing‐review & editing (equal). **Wolfgang Baumgärtner:** Conceptualization (equal); Writing‐original draft (equal); Writing‐review & editing (equal). **Eva Leitzen:** Conceptualization (equal); Methodology (equal); Writing‐original draft (equal); Writing‐review & editing (equal).

## Supporting information

Figure S1

Figure S2

Figure S3

Figure S4

Figure S5

Figure S6

Video S1

Video S2

Video S3

Video S4

Video S5

Video S6

Video S7

Video S8

Supplementary Material

## Data Availability

Data available on request from the authors.
