# Supplementary figures and images for: Phenotypical peculiarities and species‐specific differences of canine and murine satellite glial cells of spinal ganglia
Source: J Cell Mol Med. 2021 Jun 6;25(14):6909–24. doi: 10.1111/jcmm.16701 (PMC8278083; doi:10.1111/jcmm.16701)

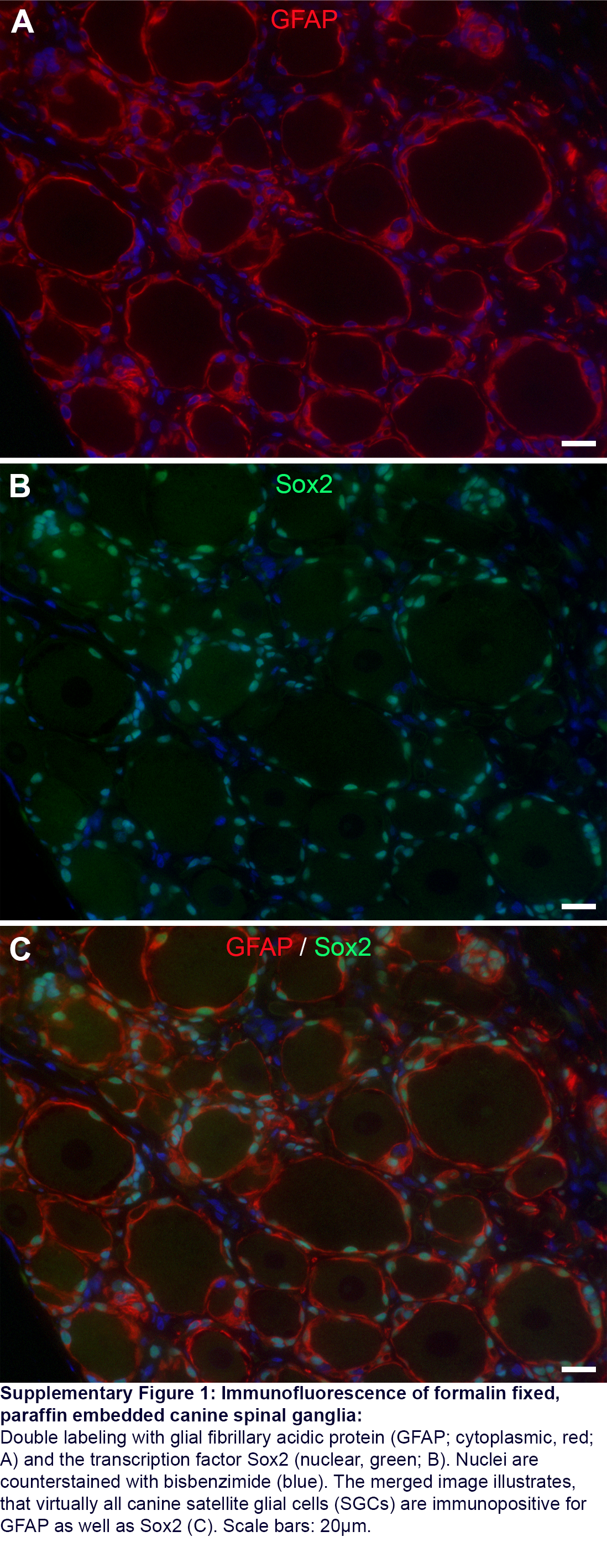

Supplement: Supplementary file 1 — Figure S1 [file JCMM-25-6909-s007.tif]

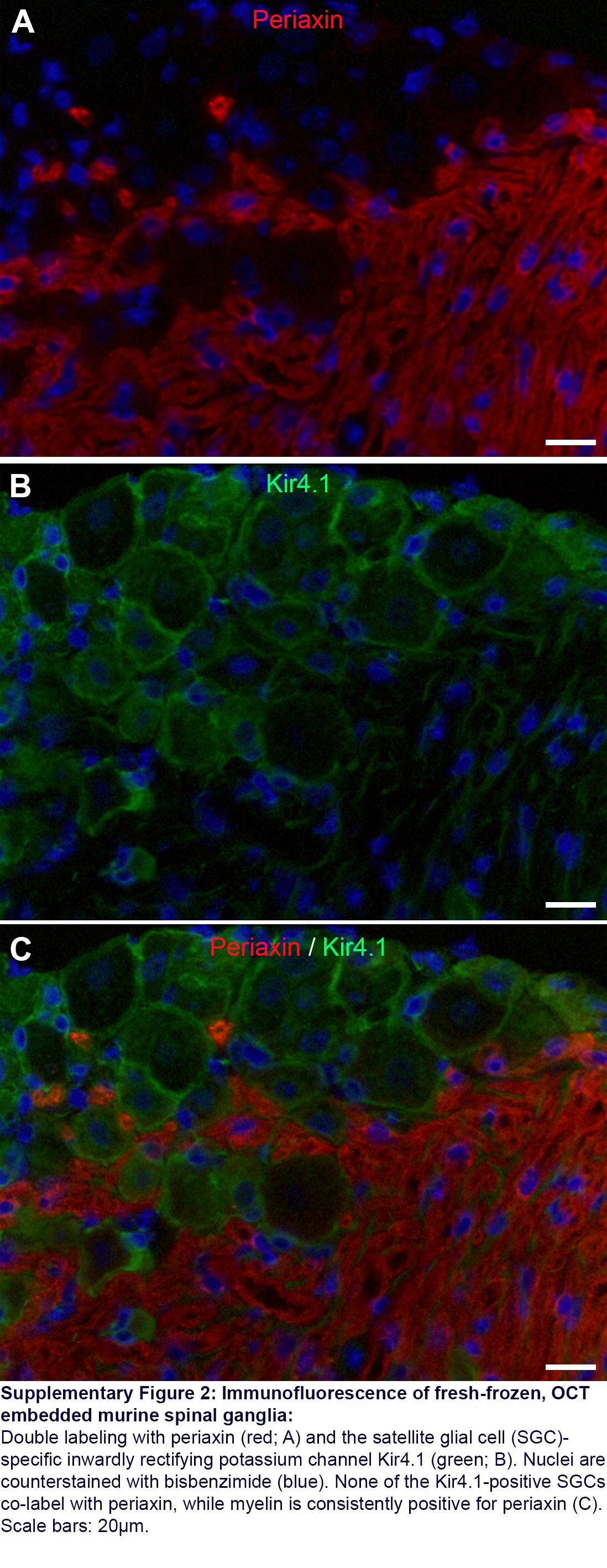

Supplement: Supplementary file 2 — Figure S2 [file JCMM-25-6909-s005.tif]

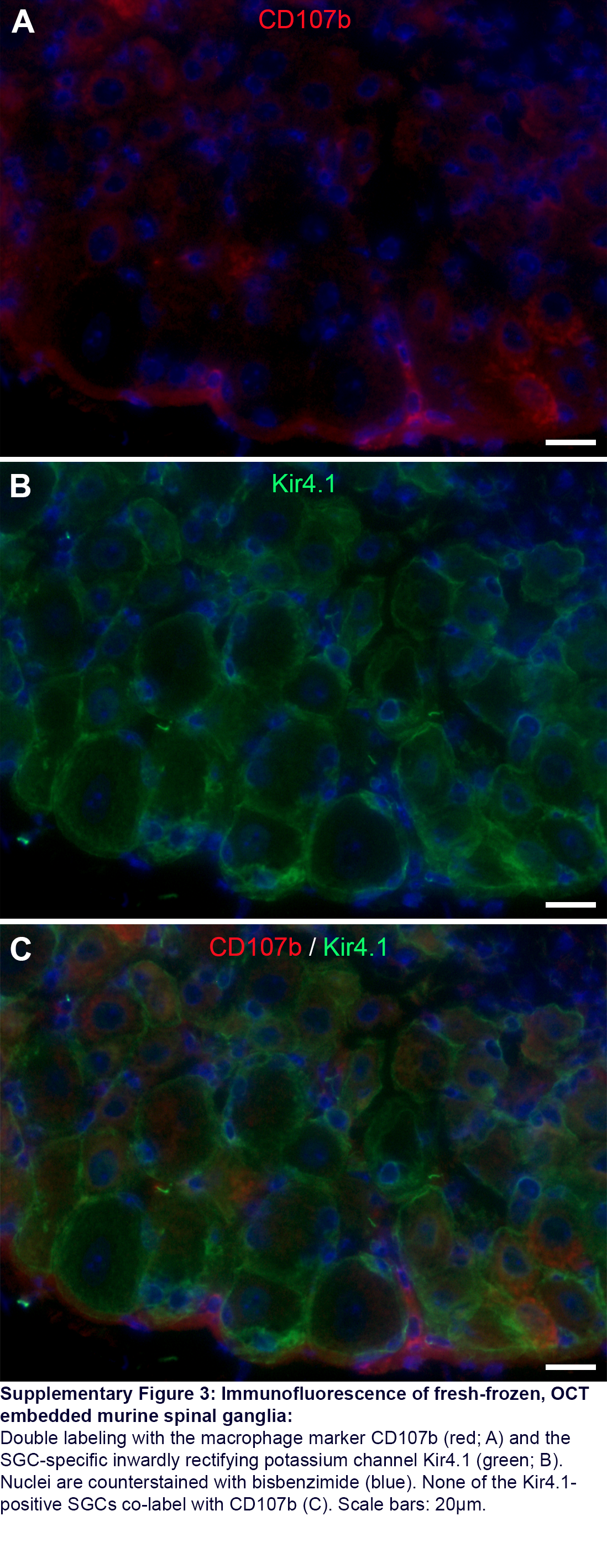

Supplement: Supplementary file 3 — Figure S3 [file JCMM-25-6909-s014.tif]

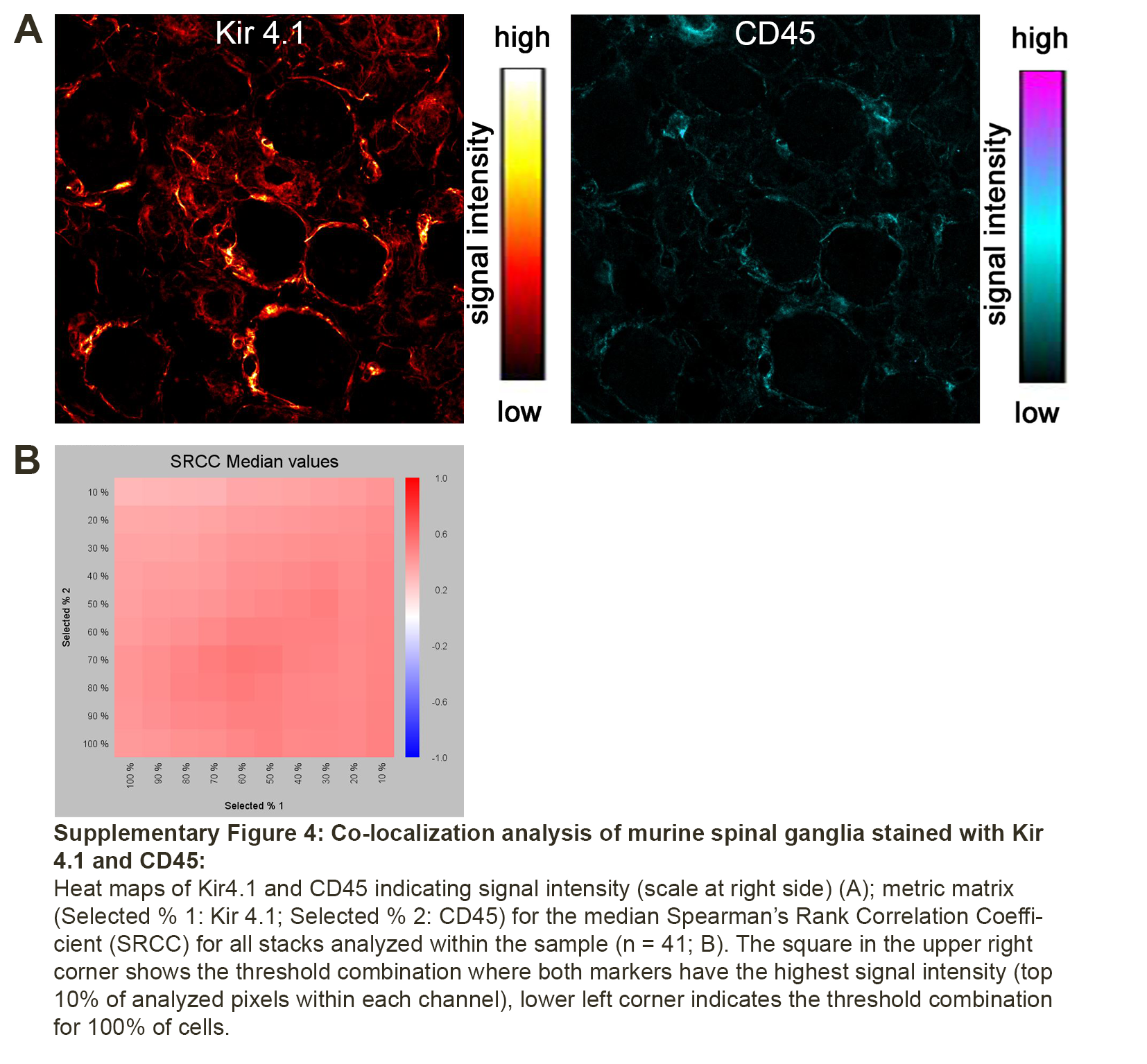

Supplement: Supplementary file 4 — Figure S4 [file JCMM-25-6909-s006.tif]

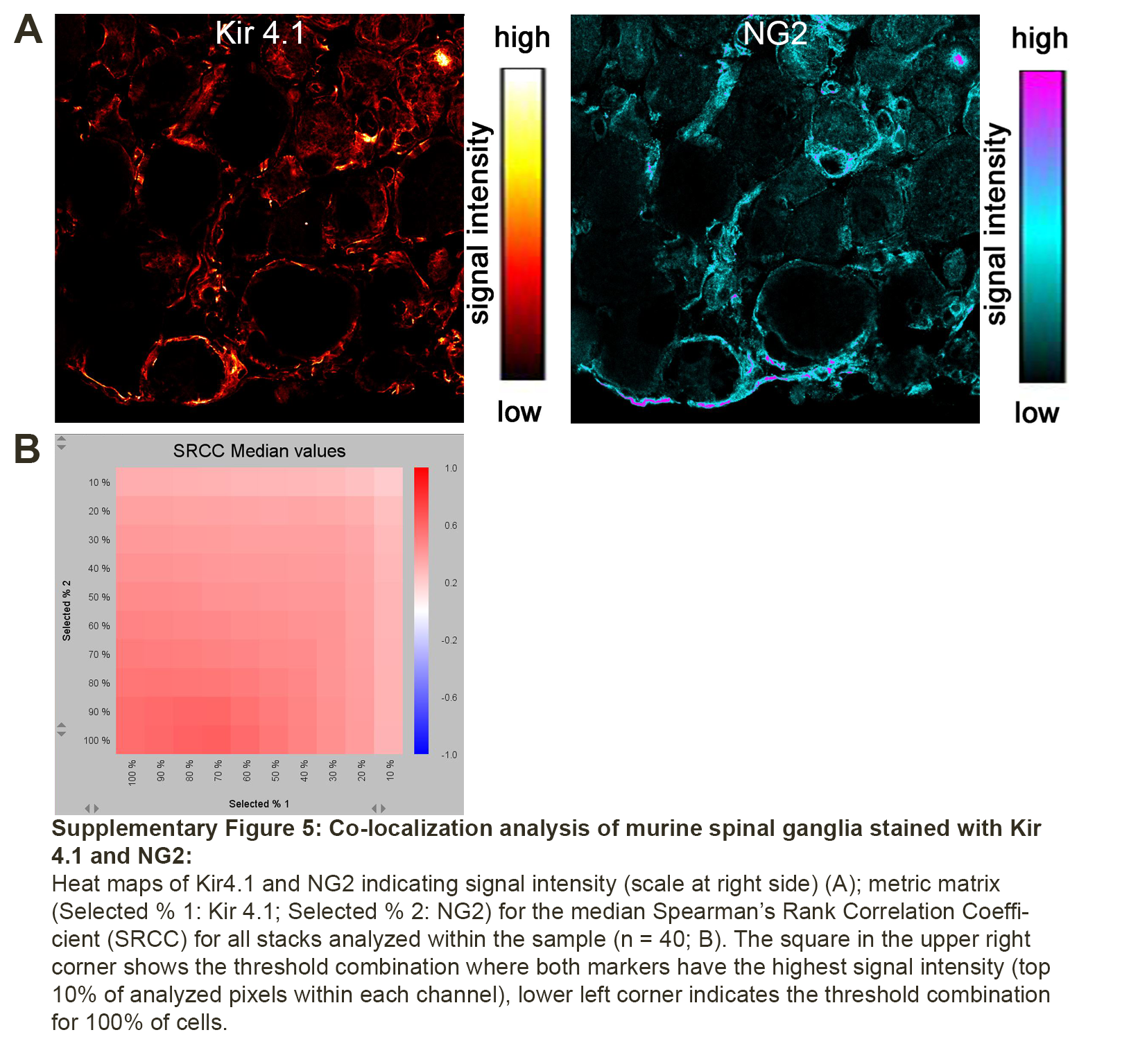

Supplement: Supplementary file 5 — Figure S5 [file JCMM-25-6909-s010.tif]

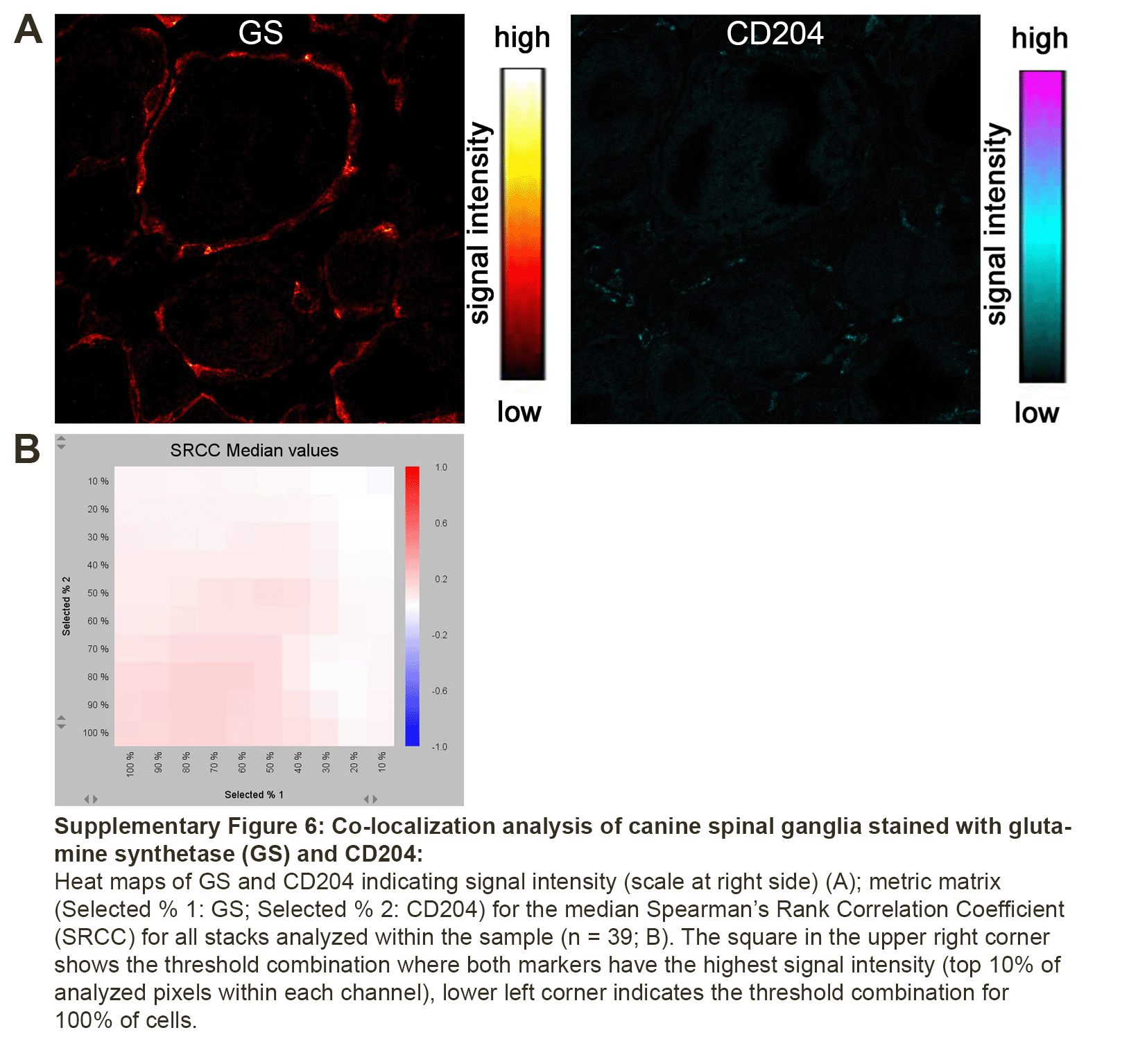

Supplement: Supplementary file 6 — Figure S6 [file JCMM-25-6909-s008.gif]
