# Supplementary material for: Phenotypical peculiarities and species‐specific differences of canine and murine satellite glial cells of spinal ganglia
Source: J Cell Mol Med. 2021 Jun 6;25(14):6909–24. doi: 10.1111/jcmm.16701 (PMC8278083; doi:10.1111/jcmm.16701)
Supplement: Supplementary file 15 — Supplementary Material [file JCMM-25-6909-s009.docx]

Supplementary Videos - Legends:

Supplementary Video 1: **Movie of 3D confocal reconstructions of Fig. 1:** Formalin fixed paraffin embedded canine spinal ganglia double labeled with glutamine synthetase (green) and the neuronal marker NeuN (magenta). Nuclei are counterstained with bisbenzimide (blue).

Supplementary Video 2: **Movie of 3D confocal reconstructions of Fig. 2:** Formalin fixed paraffin embedded murine spinal ganglia double labeled with glutamine synthetase (green) and the neuronal marker NeuN (magenta). Nuclei are counterstained with bisbenzimide (blue).

Supplementary Video 3: **Movie of 3D confocal reconstructions of Fig. 1:** Formalin fixed paraffin embedded canine spinal ganglia double labeled with the inwardly rectifying potassium channel Kir 4.1 (green) and the neuronal marker NeuN (magenta). Nuclei are counterstained with bisbenzimide (blue).

Supplementary Video 4: **Movie of 3D confocal reconstructions of Fig. 2:** Formalin fixed paraffin embedded murine spinal ganglia double labeled with the inwardly rectifying potassium channel Kir 4.1 (green) and the neuronal marker NeuN (magenta). Nuclei are counterstained with bisbenzimide (blue).

Supplementary Video 5: **Movie of 3D confocal reconstructions of Fig. 4:** Formalin fixed paraffin embedded canine spinal ganglia double labeled with the macrophage marker Iba 1 (magenta) and the satellite glial cell - specific marker glutamine synthetase (green). Nuclei are counterstained with bisbenzimide (blue).

Supplementary Video 6: **Movie of 3D confocal reconstructions of Fig. 4:** Formalin fixed paraffin embedded canine spinal ganglia double labeled with the macrophage marker CD204 (magenta) and the satellite glial cell - specific marker glutamine synthetase (green). Nuclei are counterstained with bisbenzimide (blue).

Supplementary Video 7: **Movie of 3D confocal reconstructions of Fig. 5:** Fresh-frozen, OCT embedded murine spinal ganglia double labeled with the pan-leukocyte marker CD45 (magenta) and the satellite glial cell - specific inwardly rectifying potassium channel Kir4.1 (green). Nuclei are counterstained with bisbenzimide (blue).

Supplementary Video 8: **Movie of 3D confocal reconstructions of Fig. 6:** Fresh-frozen, OCT embedded murine spinal ganglia double labeled with the the neural/glial antigen 2 (magenta) and the satellite glial cell - specific inwardly rectifying potassium channel Kir4.1 (green). Nuclei are counterstained with bisbenzimide (blue).

Supplementary Figures - Legends:

Supplementary Figure 1: **Immunofluorescence of formalin fixed, paraffin embedded canine spinal ganglia:** Double labeling with glial fibrillary acidic protein (GFAP; cytoplasmic, red; A) and the transcription factor Sox2 (nuclear, green; B). Nuclei are counterstained with bisbenzimide (blue). The merged image illustrates, that virtually all canine satellite glial cells (SGCs) are immunopositive for GFAP as well as Sox2 (C). Scale bars: 20µm.

Supplementary Figure 2: **Immunofluorescence of fresh-frozen, OCT embedded murine spinal ganglia:** Double labeling with periaxin (red; A) and the satellite glial cell (SGC)- specific inwardly rectifying potassium channel Kir4.1 (green; B). Nuclei are counterstained with bisbenzimide (blue). None of the Kir4.1-positive SGCs co-label with periaxin, while myelin is consistently positive for periaxin (C). Scale bars: 20µm.

Supplementary Figure 3: **Immunofluorescence of fresh-frozen, OCT embedded murine spinal ganglia:** Double labeling with the macrophage marker CD107b (red; A) and the SGC-specific inwardly rectifying potassium channel Kir4.1 (green; B). Nuclei are counterstained with bisbenzimide (blue). None of the Kir4.1-positive SGCs co-label with CD107b (C). Scale bars: 20µm.

Supplementary Figure 4: **Co-localization analysis of murine spinal ganglia stained with Kir 4.1 and CD45:** Heat maps of Kir4.1 and CD45 indicating signal intensity (scale at right side) (A); metric matrix (Selected % 1: Kir 4.1; Selected % 2: CD45) for the median Spearman’s Rank Correlation Coefficient (SRCC) for all stacks analyzed within the sample (n = 41; B). The square in the upper right corner shows the threshold combination where both markers have the highest signal intensity (top 10% of analyzed pixels within each channel), lower left corner indicates the threshold combination for 100% of cells.

Supplementary Figure 5: **Co-localization analysis of murine spinal ganglia stained with Kir 4.1 and NG2:** Heat maps of Kir4.1 and NG2 indicating signal intensity (scale at right side) (A); metric matrix (Selected % 1: Kir 4.1; Selected % 2: NG2) for the median Spearman’s Rank Correlation Coefficient (SRCC) for all stacks analyzed within the sample (n = 40; B). The square in the upper right corner shows the threshold combination where both markers have the highest signal intensity (top 10% of analyzed pixels within each channel), lower left corner indicates the threshold combination for 100% of cells.

Supplementary Figure 6: **Co-localization analysis of canine spinal ganglia stained with glutamine synthetase (GS) and CD204:** Heat maps of GS and CD204 indicating signal intensity (scale at right side) (A); metric matrix (Selected % 1: GS; Selected % 2: CD204) for the median Spearman’s Rank Correlation Coefficient (SRCC) for all stacks analyzed within the sample (n = 39; B). The square in the upper right corner shows the threshold combination where both markers have the highest signal intensity (top 10% of analyzed pixels within each channel), lower left corner indicates the threshold combination for 100% of cells.
